# Hospital and outpatient models for Hematopoietic Stem Cell Transplantation: A systematic review of comparative studies for health outcomes, experience of care and costs

**DOI:** 10.1371/journal.pone.0254135

**Published:** 2021-08-12

**Authors:** Marino J. González, Elena Urizar, Maider Urtaran-Laresgoiti, Roberto Nuño-Solinís, Esther Lázaro-Pérez, Lourdes Vázquez, Maria Jesús Pascual-Cascón, Carlos Solano, Mi Kwon, Cristina Gallego, Francesc Fernández-Avilés

**Affiliations:** 1 Deusto Business School Health, University of Deusto, Bilbao, Spain; 2 Faculty of Health Sciences, Valencian International University, Valencia, Spain; 3 Hematology Service, Hospital Clínico Universitario de Salamanca, Salamanca, Spain; 4 Hematology Department, BMT Unit. Hospital Regional of Málaga, Málaga, Spain; 5 Department of Medicine, Hematology Service, Hospital Clínico Universitario-INCLIVA, School of Medicine, University of Valencia, Valencia, Spain; 6 Department of Hematology, Hospital G. U. Gregorio Marañón, Institute of Health Research Gregorio Marañón, Madrid, Spain; 7 Hematology Department, At-home and Stem Cell Transplantation Unit, Institute of Hematology and Oncology, Hospital Clinic, Institut d’Investigacions Biomèdiques August Pi I Sunyer (IDIBAPS), Barcelona, Spain; European Institute of Oncology, ITALY

## Abstract

The number of Hematopoietic Stem Cell Transplantations has risen in the past 20 years. The practice of outpatient Hematopoietic Stem Cell Transplantation programs is increasing in an attempt to improve the quality of patient care and reduce the demand for hospital admission. A systematic review of 29 comparative studies between in-hospital and outpatient treatment of Hematopoietic Stem Cell Transplantation, with no restriction by outpatient regime was conducted. This study aims to analyse the current evidence on the effects of the outpatient model on patient-centred outcomes, comparing both in-hospital and outpatient models for autologous and allogeneic HSCT using the Triple Aim framework: health outcomes, costs and experience of care. We found evidence on improved health outcomes and quality of life, on enhanced safety and effectiveness and on reduced overall costs and hospital stays, with similar results on overall survival rates comparing both models for autologous and allogeneic patients. We also found that the outpatient Hematopoietic Stem Cell Transplantation is a safe practice as well as less costly, it requires fewer days of hospital stay both for autologous and allogeneic transplantations. Under a situation of an increasing number of transplants, rising healthcare costs and shortages of hospital capacity, incorporating outpatient models could improve the quality of care for people requiring Hematopoietic Stem Cell Transplantation programs.

## Introduction

Hematopoietic Stem Cell Transplant (HSCT) involves the administration of hematopoietic stem cells in patients with dysfunctional or depleted bone marrow. The two major transplant approaches are the autologous (using the patient’s own hematopoietic stem cells), and the allogeneic ones (using related- or unrelated-donor hematopoietic stem cells) [1]. HSCT practice is a highly specialized and resource-intense medical procedure [2,3]. Especially allogeneic-HSCT is associated with higher occurrence of adverse events such as graft vs. host disease (GVHD), higher incidence of acute renal failure and infectious events [4,5].

The number of HSCTs has increased constantly in the past 20 years, mainly due significant changes in the practice of HSCT. These changes included new anti-microbial agents, wider use of graft and donor types, indications in allogeneic HSCT such as genetic disorders or immunodeficiencies, autologous transplantation for autoimmune diseases, new GVHD prophylaxis and therapeutic strategies, and increased use of biomarkers to both diagnose and guide GVHD therapy. Some authors have shown the number of HSCT patients treated has grown from 4,751 to 17,155 (an increase of 360%) and from 12,199 to 23,945 (an increase of 196%) with allogeneic and autologous HSCT, respectively [6].

To date, most of the high dose therapy and the subsequent supportive care while awaiting hematopoietic recovery have been entirely performed in hospital settings, with a stay of approximately 14 days for autologous HSCT and 30 days for allogeneic HSCT. This can cause a decline in their functional capacity and exposure to nosocomial infections, especially relevant for allogeneic transplant patients when receiving immunosuppression [7,8]. Furthermore, recent studies show that more than half of total charges billed per HSCT correspond to hospital admission costs [9], with its important effect on health care systems.

In a context of a burgeoning incidence of onco-hematological and neurodegenerative diseases, and restricted healthcare resources [10,11], new care and treatment approaches have been tested. The outpatient alternative model of care, is a healthcare modality that administers specialized medical care to patients in their homes, for illnesses that would usually require hospitalization. Previous studies have shown that the outpatient model makes little or no difference in mortality and readmission rates, and that it improves patients’ experience of care, previous studies have shown that the outpatient model makes little or no difference in mortality and can avoid hospital readmission, reducing both clinical and economic burdens [7,12–14].

Since it was first reported by Jagannath et al. in 1997 [15], the outpatient model in the practice of HSCT has also been recognized for its positive results in terms of effectiveness and safety [15–19]. Improvement of anti-microbial prophylaxis and therapy, prevention of oral mucositis, the use of reduced intensity conditioning regimens and high-resolution HLA typing together with the development of ambulatory bone marrow transplantation (HSCT) units have enabled the establishment of outpatient HSCT programs [19].

Their main advantages include shorter hospital stays, lower risk of nosocomial infections, and increased comfort for patients [7,17]. Nevertheless, associated risks with outpatient modalities have been reported in the literature, such as the high frequency of hospital readmissions due to fever and infections related to neutropenia [17,19], as well as the higher occurrence of adverse events, such as graft vs. host disease (GVHD), [19].

Although numerous research has focused on studying the impact of outpatient model in HSCT [7,20], to the best of our knowledge, overall comparative effects have not yet been systematically reviewed. This study aims to analyse the current evidence on the effects of the outpatient model on patient-centred outcomes, comparing both in-hospital and outpatient models for autologous and allogeneic HSCT using the Triple Aim framework: health outcomes, costs and experience of care. In doing so, we aim to provide an overview of the body of literature published until May 2020. As far as we know, this is the most comprehensive systematic review on this topic that seeks to understand the impact of the outpatient model in HCST from the public, patient, provider and payer perspective.

Although autologous and allogeneic transplants correspond to different types according to clinical characteristics and treatments, a general perspective has been prioritized in this review, with the purpose of identifying more specific lines of comparison so that they can be continued in future studies. The results of this review may be useful in the design of controlled clinical studies aimed at comparing HSCT alternatives, as well as to advance the comparability of methods for estimating health outcomes and costs. From the perspective of health management, the results of this review can be used as a reference to explore innovative options in treatments that require HSCT. For the purpose of uniformity, in the text we use outpatient to refer to all the alternatives that identify this type of outpatient treatment.

## Methods

A systematic review to identify studies comparing outpatient and hospital HSCT care models was conducted. The Preferred Reporting Items for Systematic Reviews and Meta-Analyses (PRISMA) was used to report our findings [21].

### Data sources and searches

The search was conducted in the PUBMED and Google Scholar databases, with no time or language restrictions. The search for published articles was made using a combination of appropriate keywords, MeSH and non- MeSH index terms. The search strategy details are available in S1 File. The last update was carried out on May 17^th^, 2020. We did not apply any previous protocol.

### Inclusion and exclusion criteria

We looked for comparative studies between outpatient and in-hospital, with two different groups of patients undergoing autologous and/or allogeneic HSCT. We sought models of care that aimed to avoid or reduce hospitalisation duration under one of the following categories: 1) admission avoidance programs that provided active treatment in the patient’s home or community houses; 2) schemes that facilitated early discharge from hospital; and 3) patients receiving care in an outpatient setting and avoiding admission to hospital. No filter by medical condition or diagnoses for HSCT was considered. We included all outpatient regimes. 4) Only comparative studies with the existence of a control group were elegible. 5) Reported outcomes on at least one of the dimensions of the Triple Aim framework [22]: health outcomes, experience of care, and costs. The exclusion criteria were: 1) studies focusing on pediatric patients; 2) non primary studies, excluding: systematic reviews and meta-analysis, books, theses, PhD dissertations, conference articles, and working papers.

### Study selection

Two authors independently screened titles and abstracts for eligibility followed by full-text review for inclusion. The articles were reviewed and disagreements resolved through discussion and/or involvement of a third researcher. The initial database search generated 149 references. These were assessed for relevance based on the title, and when the information provided in the title was inconclusive, the abstract was consulted. After title and abstract revision, 95 references were identified as relevant to full -text review against the inclusion and exclusion criteria. During full-text screening, 66 studies were identified as not suitable and were discarded. The authors agreed on more than 80% of the included papers, a third reviewer was consulted for discrepancies, leaving 29 included studies in this paper. The PRISMA Flow Diagram is available in S1 Fig and the PRISMA checklist in S2 Checklist.

### Data extraction

Details pertaining to author, publication year, country, study characteristics and design, study sample, outpatient interventions scheme, and reported outcomes were extracted using Microsoft Excel.

### Data synthesis and presentation

We applied the Triple Aim [22] framework to sort results in each of its three categories. Since clinical characteristics of autologous and allogeneic HSCT cases differ, data on outcomes were classified separately for each. We presented the most frequently reported Triple Aim framework outcomes in the included papers.

## Results

Of the 149 peer-reviewed studies, 29 studies were included: 17 for autologous transplants, 9 for allogeneic transplants and two publications covering both [4,23], which are reported in both tables. One study did not specify type of transplant [24], and it was classified within the allogeneic group (Tables 1 and 2).

**Table 1:** Characteristics of studies focused on autologous transplants.

First Author	Year	Country	Study design	Outpatient regime	Total patients (n)	Inpatient care Model patients (n)	Outpatient care patients (n)
Jagannath	1997	USA	Multi Center Case Control Comparison	Outpatient Clinic	251	160	91
Meisenberg	1998	USA	Single Center Prospective Case Control Comparison	Outpatient Clinic		20	28 (46 partial)
Herrmann	1999	Australia	Single Center Prospective Case Control Comparison	Outpatient Clinic	139	88	51
Summers	2000	Canada	Multi Center Observational Study	Outpatient Clinic	41	20	21
Frey	2002	USA	Single Center Prospective Trial	Outpatient Clinic	47	26	21
Fernández-Avilés	2006	Spain	Single Center Prospective Case Control Comparison	At home	100	50	50
Stiff	2006	USA	Single Center Retrospective Case Control Comparison	Outpatient Clinic	132	32	100
McDiarmid*	2010	Canada	Single Center Retrospective Case Control Comparison	Outpatient Clinic	671	163	508
Faucher et al	2012	France	Multi Center Randomized Study	Outpatient Clinic	95	65	30
Holbro et al	2013	Canada	Single Center Retrospective Study	Outpatient Clinic	180	89	91
Graff	2015	USA	Single Center Retrospective Cohort Study	Outpatient Clinic	230	135	95
Paul	2015	USA	Single Center Retrospective Case Control Comparison	At home	301	219	82
Cantu-Rodriguez*	2016	Mexico	Observational,longitudinal, and prospective study	Outpatient Clinic	25	6	19
Abid	2017	Singapore	Single Center Prospective Case Control Comparison	Outpatient Clinic	21	11	10
Shah	2017	USA	Single Center Retrospective Study	Outpatient Clinic	1,046	669	377
Obiozor	2017	USA	Single Center Retrospective Study	Outpatient Clinic	3 groups	273	175
Martino	2017	Italy	Single Center Activity Based Costing Analysis	Outpatient Clinic	ND	ND	ND
Martino	2018	Italy	Prospective Observational Longitudinal Cohort Study	Outpatient Clinic	140	76	64
Dunavin	2020	USA	Multi Center Retrospective Cohort Study	Outpatient Clinic	1,640	1,445	195

Source: Compiled by authors based on included references.

*: This article analyses both allogeneic and autologous,

ND: Not determined.

**Table 2:** Characteristics of studies focused on allogeneic transplants.

First Author	Year	Country	Study design	Outpatient regime	Total patients (n)	Inpatient care model patients (n)	Outpatient care HaH patients (n)
Rizzo **	1999	USA	Nonrandomized prospective cohort study	Outpatient Clinic	132	115	17
Svahn	2000	Sweden	Single Center Prospective Case Control Comparison	At home	33	11	22
Svahn	2005	Sweden	Single Center Prospective Case Control Comparison	At home	90	54	36
Nicolau	2007	Brazil	Single Center Retrospective Case Control Comparison	Outpatient Clinic	100	49	51
Svahn	2008	Sweden	Single Center Prospective Case Control Comparison	At home	152	76	76
McDiarmid*	2010	Canada	Single Center Retrospective Case Control Comparison	Outpatient Clinic	392	196	196
Ringden	2013	Sweden	Single Center Retrospective Case Control Comparison	At home	292	146	146
Granot	2015	EUA	Single Center Prospective Case Control Comparison	Outpatient Clinic	1,037	548	489
Cantu-Rodriguez *	2016	Mexico	Single Center Retrospective Case Control Comparison	At home	32	19	13
Lisenko	2017	Germany	A retrospective single-centre analysis	Outpatient Clinic	128	65	63
Guru	2019	USA	Single Center Retrospective Case Control Comparison	Outpatient Clinic	151	116	35
Gutiérrez-García	2020	Spain	Single Center Retrospective Case Control Comparison	At home	80	39	41

Source: Compiled by authors based on included references

*: This article analyses both allogeneic and autologous

**: Does not specify type of transplant.

From the analysed papers, 57% of autologous papers and 90% of allogeneic analysed reported on at least one health outcomes, the most frequent were: overall survival, progression free survival and mortality rates. Concerning experience of care, safety and effectiveness outcomes were the most reported ones, in 63% and 83% of the autologous and allogeneic studies respectively. However, patient satisfaction only appeared in three studies out of the total 29. Lastly, data were also found on hospitalisations days and overall costs, in 14 autologous studies and 12 allogeneic studies (Tables 3 and 4).

**Table 3:** Summary of the Triple Aim dimensions tackled on autologous studies.

First Author	Year	Country	*Health* outcomes	*Experience of care*		*Cost*	
				Safety and Effectiveness	Patient satisfaction	Cost	Hospital Stay
Jagannath	1997	USA		x		x	x
Meisenberg	1998	USA	x			x	x
Herrmann	1999	Australia		x			
Summers	2000	Canada	x				
Frey	2002	USA	x	x		x	
Fernández-Avilés	2006	Spain	x	x	x	x	x
Stiff	2006	USA		x			
McDiarmid	2010	Canada					x
Faucher	2012	France	x			x	x
Holbro	2013	Canada	X		x	x	
Graff	2015	USA	X	x			x
Paul	2015	USA	x	x		x	x
Cantu-Rodriguez	2015	Mexico	x				
Abid	2017	Singapur	x	x		x	x
Shah	2017	USA	x			x	
Obiozor	2017	USA		x			
Martino	2017	Italy				x	
Owattanapanich	2018	Various		x			
Martino	2018	Italy	x	x			
Koo	2019	USA		x			
Dunavin	2020	USA				x	

Source: Compiled by authors based on included references.

*: This article analyses both allogeneic and autologous,

**: This article does not specify type of transplant.

**Table 4:** Summary of Triple Aim dimensions tackled on allogeneic studies.

First Author	Year	Country	*Health outcomes*	*Experience of care*		*Cost*	
				Safety and Effectiveness	Patient satisfaction	Cost	Hospital Stay
Rizzo **	1999	USA		x		x	
Svahn	2000	Sweden					
Svahn	2005	Sweden	x	x			
Nicolau	2007	Brazil	x	x			x
Svahn	2008	Sweden	x	x			
McDiarmid *	2010	Canada	x	x			x
Ringden	2013	Sweden	x	x			
Granot	2015	USA	x	x			
Cantu-Rodriguez *	2015	Mexico	x				
Lisenko	2017	Germany	X	x			x
Guru	2019	USA	x	x		x	
Gutiérrez-García	2020	Spain	x	x	x	x	x

Source: Compiled by authors based on included references.

*: This article analyses both allogeneic and autologous,

**: This article does not specify type of transplant.

### Result analysis according to the Triple Aim framework

Even though, patient selection can affect differences in mortality after 100 days time frame, autologous patients results show that mortality rates without recurrence of the disease at one year are similar when comparing inpatient and outpatient HSCT [25]. Likewise, greater survival at two [26] and four years [8] has been found in the outpatient model. Similar overall survival rates have also been reported [16,17,27,28] (Table 5).

**Table 5:** Summary of results of health outcomes for autologous HSCT patients.

First Author	Year	Nonrelapse mortality in 1 year (NMR)	Transplant related mortality (TRM)	Two year progression free-survival (PFS)	Overall Survival 1 year (OS1)	Overall Survival 2 year (OS2)
Meisenberg	1998				Similar (witout years)	
Frey	2002				Similar in both: 3 years	
Fernández-Avilés	2006				Similar in both: 3 years	
Faucher	2012				Similar in both: 10 years	
Graff	2015	Out: 0% In: 1.5%, (NS)		Out: 62% In: 54%, (NS)	Out:97% In: 91%, (NS)	Out: 83% In: 80%, (NS)
Paul	2015	Out: 0% vs In: 1.8% 100 days				
Shah	2017					Out: greater (Sig)

Source: Compiled by authors based on included references.

*NRM: Non Relapse Mortality,

TRM: Transplant Related, Mortality, OS: Overall Survival, PFS: Progression Free Survival, NS: Non Significative and Sig: Significant.

For allogeneic patients (Table 6), the rates of mortality without recurrence of the disease at one year are similar when comparing inpatient and outpatient models [5,19,29,30] for allogeneic HCST. Lower mortality at 100 days time [4] and at 5 years time [31] has also been reported. Greater survival at four years (8), and statistically significant longer survival at five years has also been found in the outpatient model [31].

**Table 6:** Summary of results of health outcomes for allogeneic HSCT patients.

First Author	Year	Nonrelapse mortality in 1 year (NMR)	Transplant related mortality (TRM)	One year progression free-survival (PFS)	Overall Survival 1 year (OS1)
Svahn	2005		Out: 13% In: 44% (Sig)		Out: 63% In: 44%, (Sig). At four years’ time
Nicolau	2007	Similar in both groups			
Svahn	2008				Out 65% In: 47% (Sig). At five years
McDiarmid	2010	Out: 14.1%, lower (Sig). 100 days			
Ringden	2013	Similar in both groups			Out: 61% In: 49%, (NS). At five years’ time
Granot	2015	Out:13% In: 26%, (Sig). At five years time			
Guru	2019	Out: 3.2% In: 10.8%, (NS)		Out: 63.6% In: 64.4%, (NS)	Out: 82.8% In: 73.8%, (NS). 1 year is assumed
Gutiérrez-García	2020	Similar in both groups			Similar in both groups

Source: Compiled by authors based on included references.

*NRM: Non Relapse Mortality,

TRM: Transplant Related, Mortality, OS: Overall Survival, PFS: Progression Free Survival, NS: Non Significative and Sig: Significant.

With respect to quality of life results, in the autologous studies, the psychological, physical, social and financial well-being has been reported with higher scores in the outpatient model [17].

In parallel, other studies have indicated that the QoL is rather similar for both care models [27,32,33]. Summers [34], reported there were no differences between the groups at any of the time intervals after transplant, and for both groups QoL was rated lowest at day 4–6, with improvements at day 12–16. In the allogeneic cases, quality of life measurements have also been reported to be similar in patients who underwent outpatient HSCT compared to inpatient HSCT after three months [23].

### Experience of care

Incidence of neutropenic fever, mucositis, cumulative Graft versus Host Disease (GVHD), as well as median time to neutrophil recovery and median time to platelet recovery are some of the recurrent reported variables (Tables 7 and 8).

**Table 7:** Summary of safety and effectiveness results for autologous HSCT patients.

First Author	Year	Neutropenic Fever	Fever	Infections	Mucositis	Neutro recove	Platelet reco	Non-hema tox	Time of engraftment	Karnofsky Performance Status
Jagannath	1997	NS								
Herrmann	1999	Similar		Out: No septic shock. Less infections (NS)						
Frey	2002			NS	NS					
Fernández-Avilés	2006	Out: 76%, In: 96% (Sig)	Out: 2 days, In: 6 days (Sig)		NS					
Stiff	2006				NS				Equal	
Graff	2015	Similar		NS		Out: 10 days, In: 11 days, (Sig)	Out: 19 days, In: 20 days, (NS)	29% (both)		
Paul	2015			Out: 22%, In 46% (Sig)						
Abid	2017	NS								
Obiozor	2017									Out: greater
Martino	2018				NS					

Source: Compiled by authors based on included references.

*GVHD = Graft versus Host Disease,

NS = Non Significative, Sig = Significant, In = Inpatient and Out = Outpatient.

**Table 8:** Summary of safety and effectiveness results for allogeneic HSCT patients.

First Author	Year	Neutropenic Fever	Infections (I) or Bacteremia (B)	Mucositis	RPN	Incidence GVHD	Cumulative GVHD	Days to discharge Out clinic	Oral nutrition
Rizzo	1999					NS			
Svahn	2000	30			Out: 3 days, In: 24 days, median, (Sig)	NS		Out: 20, In: 35 median (Sig)	Out: Less days of parenteral nutrition (Sig)
Svahn	2005	32	(B) Out: 3 patients In: 9 patients, (Sig)			Out: 17%, In: 44% in stage II-IV (Sig)	Out: 52%, In: 57%		
Nicolau	2007					NS			
Svahn	2008	35					Out: lower probabiliy of GVHG grade II and IV		Out: greater
McDiarmid	2010		(I) Out: Less difference (Sig)						
Ringden	2013						Out: 15% outpatient Others: 32–44%		
Lisenko	2017	Out: 57%, In: 86% (NS)		NS					
Guru	2019	Out: 8,5%, In: 25,8% (sig)	(I) NS	Out: 8,5%, In: 50,8% (Sig)	Out: 5,7%, In: 20,6; (Sig)	NS	Out: 25,7%, In: 25,2% (grades II to IV), Out: 8,5%, in: 10,4 (III to IV), Out: 51,6%, in: 38,3 (chronic)		
Gutiérrez-García	2020	Out: 32%, In 90% (sig)		NS			Out: 10%, In: 29% (Sig)		

Source: data published on reviewed articles.

*GVHD = Graft versus Host Disease,

RPN = Requiered Parenteral Nutrition, NS = Non Significative, Sig = Significant, In = Inpatient and Out = Outpatient.

For autologous patients the frequency of infections has been reported to be lower (with statistical significance) in the outpatient model [4,9], although similar frequency of infections has also been reported in some papers for both models of care [25,27].

In particular, the frequency of neutropenic fever has been shown to be lower in the outpatient option [17]. A similar frequency has also been reported in both options [25,35]. Frequency of mucositis has also been reported to be similar when comparing inpatient and outpatient HSCT [17,25,27,32].

In allogeneic HSCT, the frequency of neutropenic fever has been shown to be lower in the outpatient option [19,30]. In all the works except in [36], the differences were significant. The lowest frequency of neutropenic fever in patients with outpatient HSTC (8.5%) was reported in [30]. Furthermore, in the outpatient HSCT model, the frequency of mucositis is shown to be six times lower than in hospitalized patients [30]. A similar frequency has also been reported when comparing inpatient and outpatient HSCT [19]. With respect to the acute incidence of transplant rejection or GVHD, it has been reported to be significantly lower in the home modality [8]. Once again, similar incidence has also been reported in the two models of care [24,29,30,37]. The higher frequency of oral nutrition in the outpatient model has been associated with lower probability of GVHD [31].

Patient satisfaction has been reported in a limited number of papers. Gutierrez Garcia et al. [19] reported that patients in hospital experience more stress than those with outpatient care, causing release of inflammatory cytokines. The outpatient care model has also received positive results [30], where the patient satisfaction score reported was 1.3 out of 6, one being excellent. Fernández-Avilés et al. [17] invited thirty patients and caregivers to complete an anonymous questionnaire after the entire procedure was completed, with 95% of them indicating that, they would choose to receive outpatient autologous HSCT again and that they would recommend the procedure to a fellow patient. Other studies [38] have also described favourable feedback from outpatient autologous patients on their experience of care.

### Cost of health care

Regarding costs, this research shows that the outpatient model is less costly than the inpatient care model. It is important to note that these results are derived from comparative studies in which at least one of the options corresponded to the treatment considered as standard.

The hospital stay is shown to be shorter in the outpatient care model for both autologous (Table 9) and for allogeneic transplants (Table 10). In autologous transplants, all studies, 8 out 8, report a reduction of the length of hospital stay in the outpatient model, ranging from a 3 days reduction, up to 17 days difference. In the allogeneic patients (Table 10), 3 out of 4 studies report a reduction of up to 11 days in hospital stay, only one study [19], reflects an increase of two additional days on average, in the outpatient model.

**Table 9:** Duration of hospital stay for autologous HSCT (in days).

First Author	Year	Inpatient (days)	Oupatient (days)	Difference
Jagannath	1997	15	9	6
Meisenberg	1998	17.3	2.7	14.6
Fernández-Avilés	2006	17	0	17
McDiarmid	2010	24	21	3
Faucher	2012	12	9	3
Graff	2015	19.2	5.4	13.8
Paul	2015	18	9	9
Abid	2017	18.3	6.9	11.4

Source: Calculations based on data reported in publications.

**Table 10:** Duration of hospital stay for allogeneic HSCT (in days).

First Author	Year	Inpatient	Outpatient	Difference
Nicolau	2007	28	17	11
McDiarmid	2010	40	35	5
Lisenko	2017	22	21	1
Gutiérrez	2020	30	32	-2

Source: Calculations based on data reported in publications.

Ten research articles were found to compare costs between outpatient and inpatient care model for autologous HSCT patients (Table 11), all of them reported a positive reduction in favour of the outpatient model, ranging from a 19.32% [28], to a 46.48% [17] reduction of cots. In the case of the allogeneic HSCT patients, two authors recently studied costs, both of them report a favourable scenario for the outpatient model, showing a 11.37% [30] and a 27.17% [19] overall cost reduction.

**Table 11:** Reduction of costs (percentage) between outpatient and inpatient care model for autologous HSCT patients.

First Author	Year	% reduction
Jagannath	1997	26.40%
Meisenberg	1998	34.32%
Frey	2002	28.37%
Fernández-Avilés	2006	46.48%
Faucher	2012	19.32%
Holbro	2013	31.36%
Abid	2017	32.72%
Shah	2017	29.70%
Martino	2017	42.34%
Dunavin	2020	43.15%

Source: Calculations based on data reported in publications

## Discussion

This systematic review of 29 comparative studies demonstrates that the outpatient model -in its different forms–have several benefits for healthcare organizations and patients. This paper is the first to comprehensively synthetize the current evidence from the Triple Aim perspective.

Pooled data from selected articles reveal that mortality rates without recurrence of the disease at one year are similar when comparing inpatient and outpatient HSCT. In the allogeneic case, the outpatient model has demonstrated a significantly lower mortality at 100 days [4] and at 5 years [39], and a significantly lower transplant-related mortality [8]. These results conform with the existing literature, having Ritchie [40] found that outpatient HSCT was not associated with increased morbidity.

Analysis on the QoL revealed no conclusive results on the impact of the outpatient model in HSCT. Significant improvement in QoL has been reported for the autologous [17] and allogeneic transplants [19,36]. However, other studies demonstrated no differences in reported QoL between both care models. This is an area that requires further research, as other authors have indicated [17]. However, there is also a need to use other data bases with publications more related to quality of life.

When looking at safety and effectiveness, the outpatient option scores higher in both autologous and allogeneic cases. Numerous studies show the frequency of neutropenic fever, the appearance of mucositis and the frequency of infections to be lower in the outpatient option [17,25,27,32], although there have also been studies reporting no differences [35]. The higher frequency of oral nutrition in the outpatient model has been associated with lower probability of side effects [29], which is in line with results from other clinical trials [41,42]. Furthermore, Owatanapach et al. [7] found that patients who underwent a HSCT in an outpatient setting actually had a significantly lower risk of developing infectious complications, including 56% reduced odds of developing febrile neutropenia and 60% reduced odds of developing septicemia.

Regarding costs, this research shows that the outpatient model is less costly than the inpatient model. According to our estimated average, the number of days of hospital stay in the outpatient model is 55% and 19% less than the hospital-based model, in the autologous and allogeneic cases, respectively. As Martino et al. explain, this leads to ease of hospital bed shortage and shorter wait times [43]. The average reduction in charges or costs with respect to the hospital-based care model is 33.42% and 19.27% for autologous and allogeneic transplants, respectively. These data on reduction of costs are highly relevant as the HSCT is a resource-intense and costly intervention. Data from Guru et al. [30], revealed the average national cost for the allogeneic HSCT ranged over 267,000 U.S. dollars in the United States. Reviews in this field also show outpatient autologous HSCT is associated with a significantly reduced bed occupancy [40].

However, there are some limitations to these findings. Firstly, there is considerable variability among studies. When looking at length of hospital stay, indications for hospitalization of outpatients differ between studies. As other investigators point out, further research should adopt common definitions of hospital-outpatient care or collect and report data on clinical components more explicitly to facilitate comparisons across models [12]. Secondly, early discharge, and comprehensive or total hospital at home programmes were all considered for the purpose of this review. As some authors suggest, this may make the comparison and generalisation of results more difficult [27,28]. Thirdly, only one randomised clinical trial was found [28], exposing several limitations for the comparison analysis due to the variability in study designs and patient selection criteria. Indicating the set of clinical variables could have been the deciding factor for clinicians when choosing an inpatient versus an outpatient strategy, which can affect the results [26,28,44]. Martino et al. [45], elaborated Italian consensus guidelines in 2016 to homogenize and bridge gaps in these aspects of outpatient HSCT. Lastly, some individual studies express their limitation in the generalizability of their cost analysis results due to context factors. It should be noted that only the costs included by the authors of the papers are reported in our analysis. A further more detailed analysis of the methodology of cost analysis can be very useful to agree on better options for cost comparison. The complexity in the definition and practice of cost analysis of hematopoietic stem cell transplantation programs has been reported by Al-Hashmi et al 2020 [46].

Considering the above limitations, we propose that future research should: firstly, take a look from a management perspective and propose homogeneous study designs and protocols for clinical trials in Hospital and outpatient models for Hematopoietic Stem Cell Transplantation (HSCT); secondly, when studying costs, these type of innovations require the consideration of multiple perspectives, beyond clinical aspects, and as such, opportunity cost and calculate cost such as staff resources, or the role of a caregiver, which are needed to implement such a new model of care and lastly, focus on making further formal assessment of the experience of care of patients, family and caregivers. There is a clear gap in gathering and publishing this knowledge which can be used to evaluate and improve the management of outpatient schemes.

This research is the first to compare the published results on HSCT between in-hospital and outpatient models in peer-reviewed journals, while bringing a unique perspective to the current body of literature, looking at the integrated impact on health outcomes, experience of care and cost. Limited by the heterogeneity among papers, this study concludes that the outpatient HSCT is safe and effective and its main advantages include significant cost reduction, decrease in length of hospitalization, alleviating constraints on chronic bed shortage, and facilitating patient convenience.

## Supporting information

S1 Checklist PRISMA 2009 checklist I. (TIF)

S2 Checklist PRISMA 2009 checklist II. (TIF)

S3 Checklist PRISMA 2009 checklist III. (TIF)

S1 Fig PRISMA diagram. (TIF)

S1 File Descriptor combination. (TIF)
