# Supplementary figures and images for: Hospital and outpatient models for Hematopoietic Stem Cell Transplantation: A systematic review of comparative studies for health outcomes, experience of care and costs
Source: PLoS One. 2021 Aug 12;16(8):e0254135. doi: 10.1371/journal.pone.0254135 (PMC8360565; doi:10.1371/journal.pone.0254135)

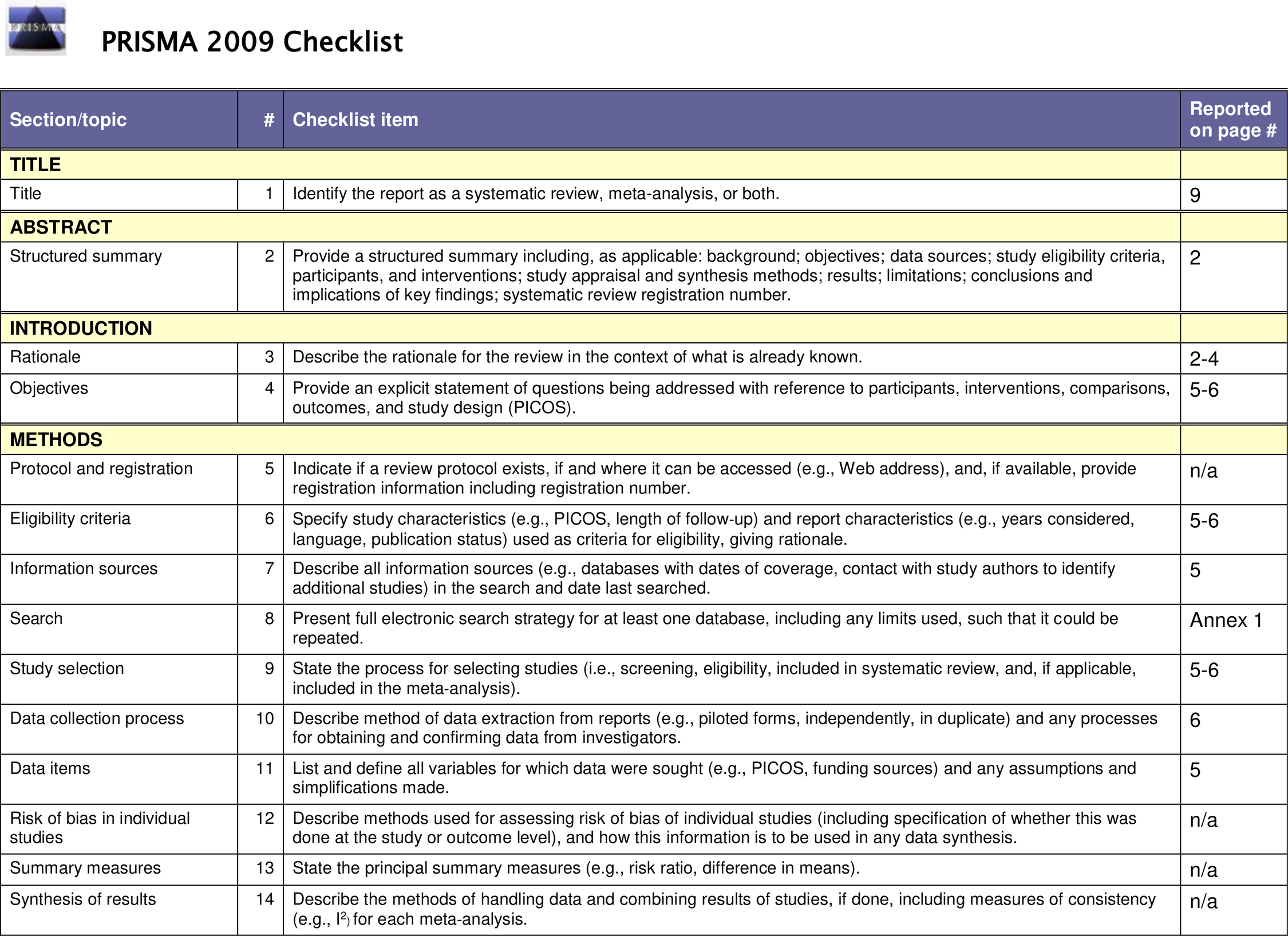

Supplement: S1 Checklist — (TIF) [file pone.0254135.s001.tif]

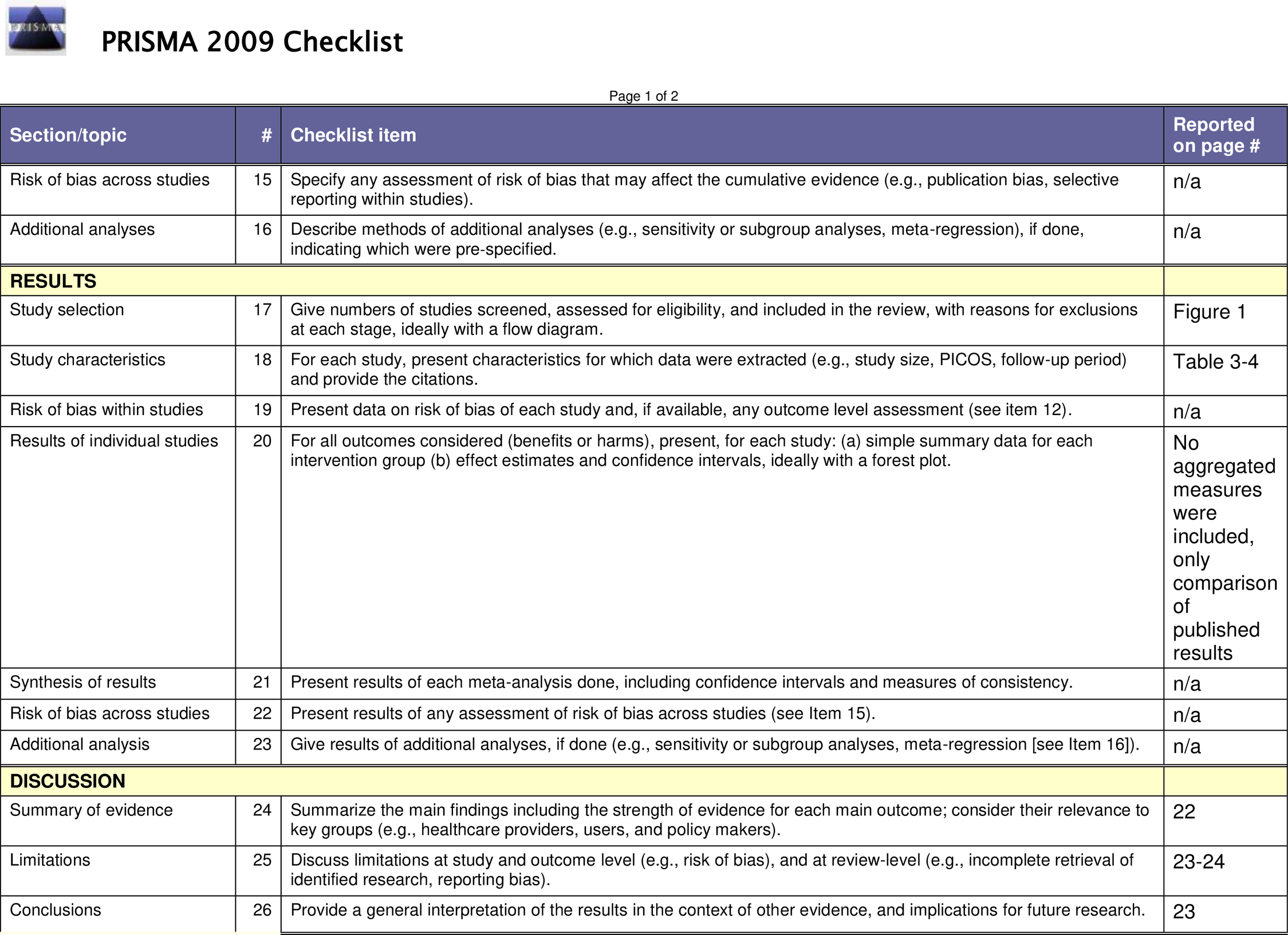

Supplement: S2 Checklist — (TIF) [file pone.0254135.s002.tif]

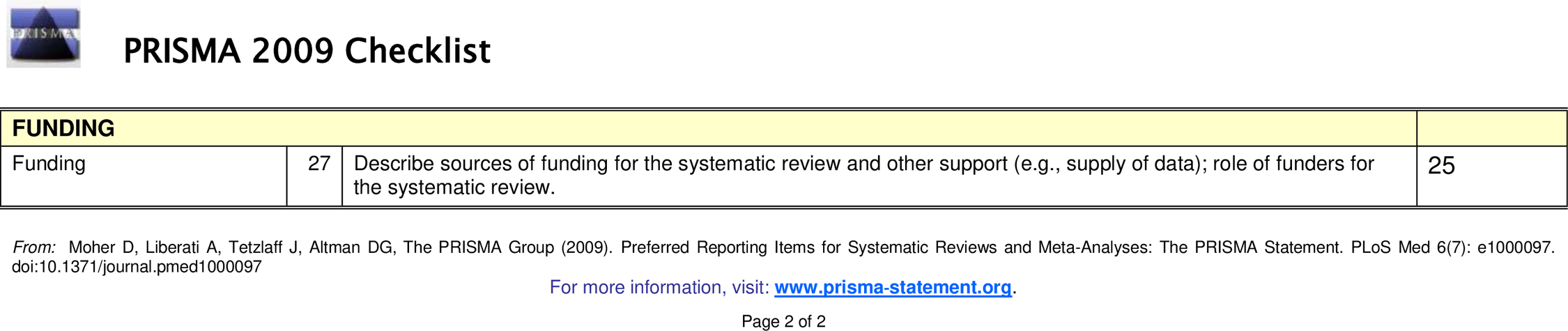

Supplement: S3 Checklist — (TIF) [file pone.0254135.s003.tif]

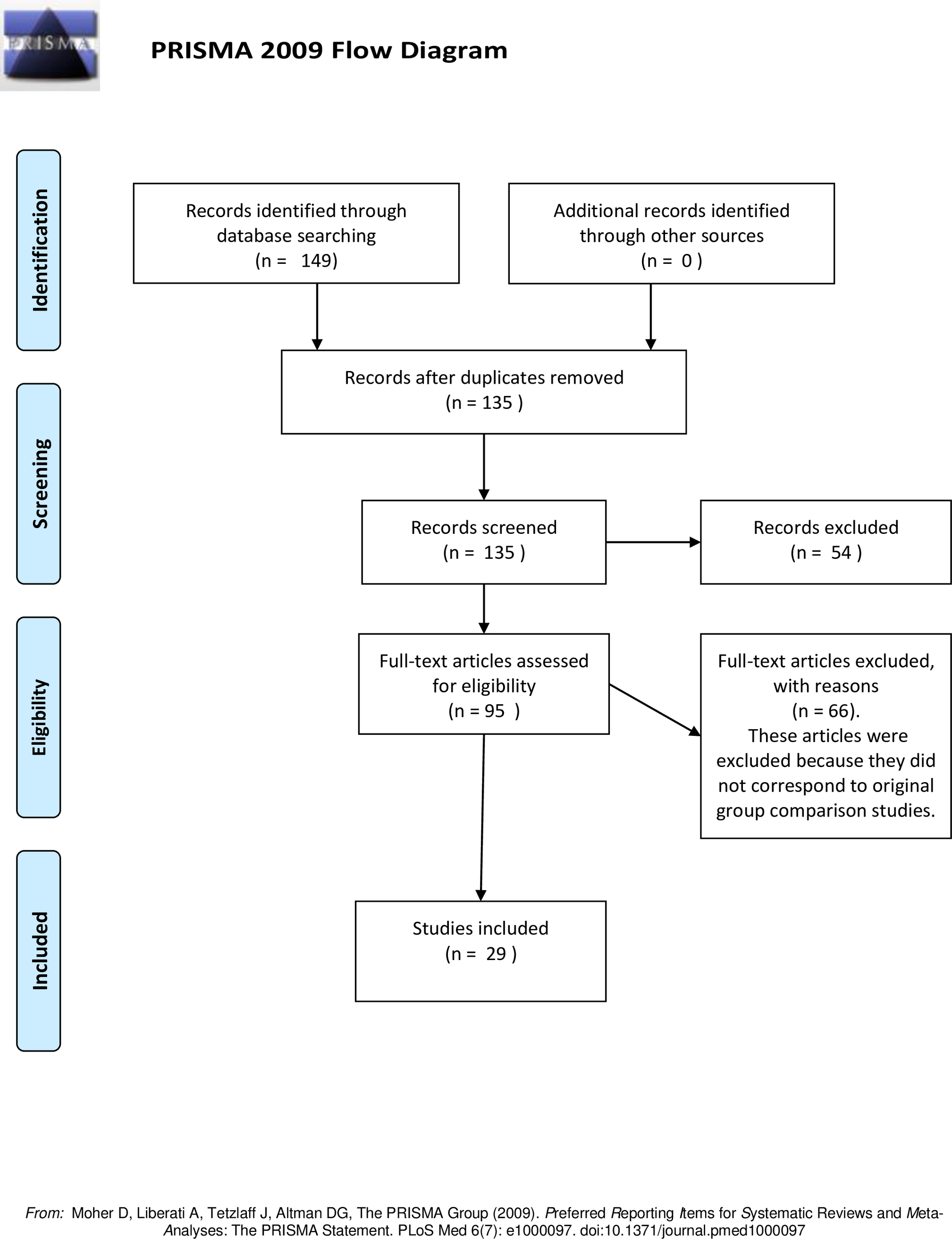

Supplement: S1 Fig — (TIF) [file pone.0254135.s004.tif]

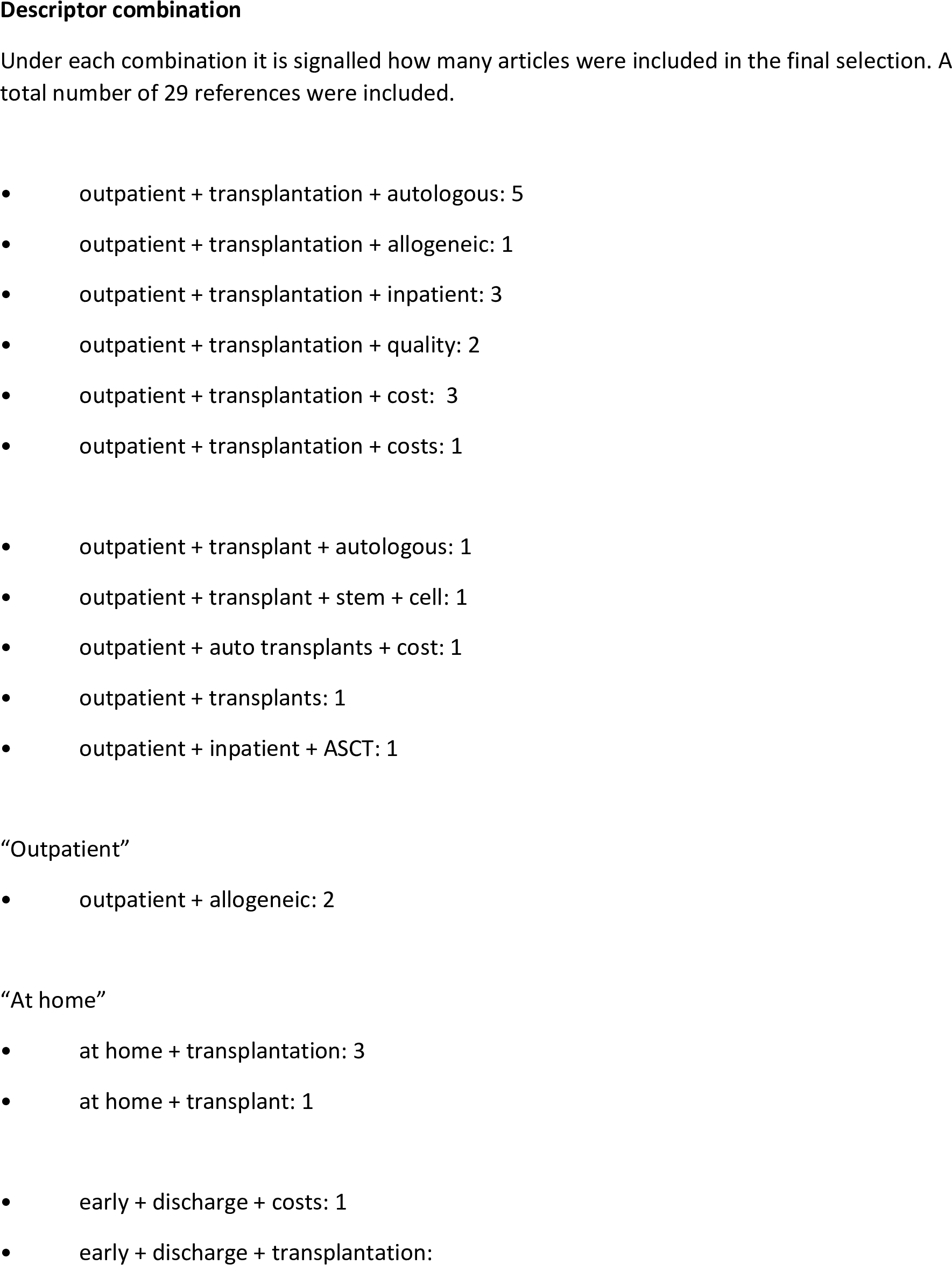

Supplement: S1 File — (TIF) [file pone.0254135.s005.tif]
